# Identification of Alzheimer's Disease-Related Genes Based on Data Integration Method

**DOI:** 10.3389/fgene.2018.00703

**Published:** 2019-01-25

**Authors:** Yang Hu, Tianyi Zhao, Tianyi Zang, Ying Zhang, Liang Cheng

**Affiliations:** ^1^Department of Computer Science and Technology, School of Life Science and Technology, Harbin Institute of Technology, Harbin, China; ^2^Department of Rehabilitation, Heilongjiang Province Land Reclamation Headquarters General Hospital, Harbin, China; ^3^Department of Bioinformatics Science and Technology, Harbin Medical University, Harbin, China

**Keywords:** Alzheimer disease, SNPs, mendelian randomization, GWAS, eQTL

## Abstract

Alzheimer disease (AD) is the fourth major cause of death in the elderly following cancer, heart disease and cerebrovascular disease. Finding candidate causal genes can help in the design of Gene targeted drugs and effectively reduce the risk of the disease. Complex diseases such as AD are usually caused by multiple genes. The Genome-wide association study (GWAS), has identified the potential genetic variants for most diseases. However, because of linkage disequilibrium (LD), it is difficult to identify the causative mutations that directly cause diseases. In this study, we combined expression quantitative trait locus (eQTL) studies with the GWAS, to comprehensively define the genes that cause Alzheimer disease. The method used was the Summary Mendelian randomization (SMR), which is a novel method to integrate summarized data. Two GWAS studies and five eQTL studies were referenced in this paper. We found several candidate SNPs that have a strong relationship with AD. Most of these SNPs overlap in different data sets, providing relatively strong reliability. We also explain the function of the novel AD-related genes we have discovered.

## Introduction

Alzheimer's disease (AD) is a neurodegenerative disease and has become the fourth major cause of death after cardiovascular disease, malignant tumor and stroke in the elderly (Senova et al., 2018). The cause of Alzheimer's disease remains difficult to explain. It is estimated that the impact of related genes, on the risk of AD, is nearly 70% (Cruchaga et al., 2012). Therefore, screening for candidate genes and loci associated with AD is of great significance to understand the pathogenesis and treatment of AD.

At present, the Genome-wide association study (GWAS) is widely used to identify various neurological susceptibility genes. The use of the GWAS analysis to find the risk genes of AD was only started 10 years ago (Visscher et al., 2012). At the beginning of this century, many research groups identified the susceptible locus of AD, but the actual results were not satisfactory. The only susceptibility genes found by these different research groups was SOL1 (Meng et al., 2007). The inconsistency of these research results were mainly due to the heterogeneity of the experimental samples, the complex linkage disequilibrium patterns, differences in the allele frequencies, and the size of the samples (Malhotra et al., 2012). Over the past 10 years, the emergence of high-throughput sequencing technology has allowed researchers to simultaneously detect millions of Single Nucleotide Polymorphisms (SNPs) on the genome. Through the efforts of large organizations and companies, high-throughput sequencing technologies have led to the discovery of many new pathways and susceptibility genes for AD in recent years. Researchers from the GWAS first identified four susceptible gene loci, CLU, PICALM, CRL, and BIN1 (Kauwe et al., 2011; Barral et al., 2012). Later, other research groups (Hollingworth et al., 2011; Naj et al., 2011) found susceptibility loci such as CD33 and EPHA1, using a larger sample of the GWAS analysis. The experimental samples of these studies were mainly from Europe. The most significant SNP population attribution scores, of the above-mentioned susceptibility loci, are between 1 and 8%, and the odds ratio (OR) varies from 1.16 to 1.20. In addition, Hillburns et al. (2014) linked neurological Parkinson's disease within the GWAS and found a range of risk locus. Kauwe et al. (2008) used the GWAS for AD research and found that risk genes such as ACE and MMP3 are associated with Aβ amyloid and Tau protein levels in cerebrospinal fluid.

However, limitations in the GWAS still remain. For example, the strategy is based on the hypothesis of “disease-common variations,” which misses rare variants [minor allele frequency (MAF < 0.005)] that may play a more important role in the cause of diseases; SNPs are not necessarily true pathogenic locus, but only “tags” SNPs that are associated with LD in real disease-causing locus. In particular, signals located in the so-called “desert regions” of genes make it difficult to elucidate the biological functions of genetic variation. The GWAS usually analyzes the marginal effects of a single locus, based on the most obvious principle of statistical discrepancies, while ignoring the interaction of multiple genes in complex diseases (Battle et al., 2014). Therefore, the GWAS still cannot fully reveal the genetic susceptibility factors of complex diseases, which is an important part of exploring genetic etiology mechanisms of complex diseases. How to mine the susceptible locus of the GWAS and how find the true pathogenic locus, as well as how to explore the biological mechanism of these non-coding sequences, is a major challenge in genetic research.

Expression quantitative trait loci (eQTL) are quantitative traits based on the expression level of the gene mRNA. Traditional QTL methods are used to locate the genetic loci and to locate the genetic locus of the target gene expression. This mapping method requires measuring the genotype and the gene expression levels for each individual, and then compares the association between the genotype and gene expression levels using an association analysis (outcrossing population) or a linkage analysis (pedigree or experimental hybridization population) (James Ronald, 2007; Clément-Ziza et al., 2015). It was found that about 80% of the genetic susceptibility loci detected by the GWAS were located in the non-coding region of the genome, suggesting that the pathogenic loci may have regulatory functions on gene expression. Nicolae et al. (Dan et al., 2010) compared the SNPs, found in the common complex diseases of the GWAS, with other random sampling SNPs with same typing platform matching the allelic frequency distribution, and found that the former clearly contained more eQTLs (Cheng et al., 2016, 2018a,b). Therefore, an important role of the large-scale eQTL research is to be able to prioritize the screening of possible pathogenic sites among SNP loci in the GWAS susceptible regions (Cheng et al., 2018c; Hu et al., 2018), and to speculate the possible biological mechanism through the impact of DNA polymorphism on biological traits. At present, many studies have used eQTL analysis as a very effective tool (Libioulle et al., 2007; Moffatt et al., 2007) to interpret the results of the GWAS. By increasing the sample size, the problem of low statistical efficiency caused by the small sample size in the past, has been gradually improved (Albert and Kruglyak, 2015), and the number of eQTLs found has significantly increased.

Therefore, if we combine the GWAS with eQTL, we will find many genes and loci related to diseases. In this paper, we introduce the “Summary Mendelian Randomization, SMR” method. This method can find disease-related SNPs from the summary level of data. SMR is first proposed in the paper by Zhu et al. (2016) as the “Integration of summary data from GWAS and eQTL studies predicts complex trait gene targets.” They used this method to identify several genes which are related to five complex traits. Since the paper was published, many researchers use SMR to identify disease-related SNPs. Pavlides et al. (2016) used the SMR in 28 GWAS datasets to identify genes with expression levels associated with traits and diseases due to pleiotropy or causality. Meng et al. (2018) applied the SMR to identify bone mineral density (BMD) related genes. Du et al. (2018) also identified genes and pathways associated with Amyotrophic Lateral Sclerosis using the SMR. Fan et al. (2017) identified six genes associated with neuroticism using the SMR. Yengo et al. (2018) identified an enrichment of eQTLs amongst lead height and BMI signals, prioritizing 684 and 134 genes, respectively, using the SMR. Liu et al. (2018) used the SMR for research on Obesity and found 20 BMI associated genes. Veturi and Ritchie (2018) compared two popular methods: The MP and SMR using different datasets. Looking at these studies and the evidence, we concluded that the SMR is an effective tool.

## Methods

### SMR

**Figure 1:**
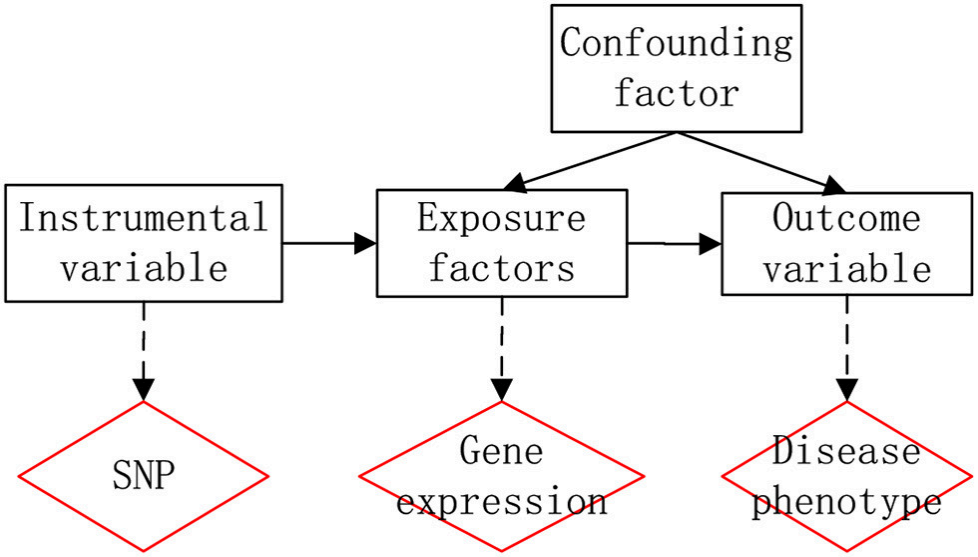
Generalization of Mendel's randomized method.

The GWAS has identified thousands of mutations associated with various traits and diseases. However, due to the complex linkage effects and statistical errors of the samples, the mechanisms and effects of these mutations on diseases remain unknown. If the level of a gene expression is affected by mutation, the level of gene expression will be different among individuals of different genotypes, therefore, if the level of expression of a gene affects the disease, then different genotypes will have different phenotypic effects on the disease. The idea is therefore very similar to Mendelian Randomization (MR), where SNPs can serve as instrumental variables to explore the association between genes and diseases. Therefore the new work flow of generalization of Mendel's randomized method is as Figure 1.

eQTL refers to regions on chromosomes that specifically regulate the expression of the mRNAs and proteins. The expression level of the mRNAs or proteins is proportional to the quantitative traits. eQTLs can be divided into cis-eQTLs and trans- eQTLs. Cis-eQTLs are the eQTLs of a gene that are located in the genomic region of the gene, indicating changes in mRNA levels that may be caused by differences in the gene itself; trans- eQTLs are the eQTLs of a gene that are located in other genomic regions, indicating other genes. These genetic differences control the different mRNA levels of the gene. The corresponding data of gene expression levels of individuals with different genotypes will then be obtained with eQTL.

**Figure 2:**
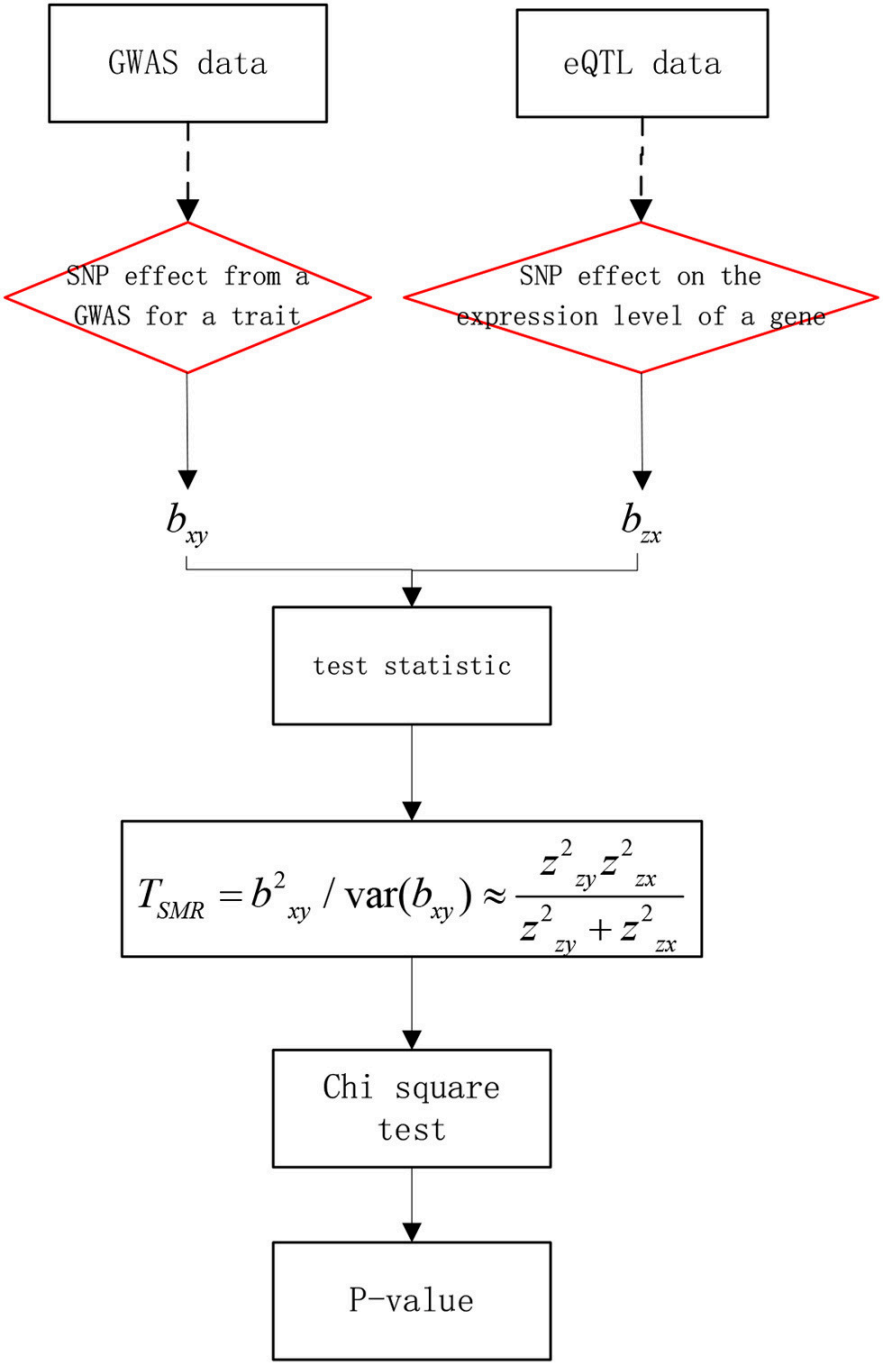
Work flow of SMR.

The basic ideas are as follows: first, set y as the phenotype (output variable), x as the expression of gene (exposure factor), and z as the gene mutation (instrumental factor). Then, *b*_*xy*_ is the effect of x on y. *b*_*zx*_ is the effect of z on x. *b*_*zy*_ is the effect of z on y. finally, *b*_*xy*_ is defined as *b*_*xy*_ = *b*_*zy*_/*b*_*zx*_, that is the effect of gene expression on the phenotype without confounding factors. The work flow of SMR is shown in Figure 2. First, *b*_*xy*_ can be obtained using the GWAS, as GWAS data can reflect the SNP effect on the trait. Then, *b*_*zx*_ can be obtained by the eQTL, as eQTL data can reflect the SNP effect on gene expression. Finally, we could use a Chi-square test to obtain significance.

## Results

As shown in Table 1, we totally got 2 GWAS datasets and 5 eQTL datasets. We combined these datasets for a total of 10 experiments. From these 10 experiments, we detected SNPs with statistical effects in five experiments. In total, we found 27 SNPs which are associated with AD.

**Table 1:** The number of SNPs obtained in five experiments.

**GWAS**	**eQTL**	**Number of SNPs**
IGAP_stage_1	CD4-cis-eQTLsFDR-ProbeLevel0.5	10
IGAP_stage_1	CD8-cis-eQTLsFDR-ProbeLevel0.6	9
AD_GERAD_GWAS_ autosomal_additive	Hap300_CER_AD	1
AD_GERAD_GWAS_ autosomal_additive	CisAssociationsProbeLevelFDR0.5	40
AD_GERAD_GWAS_ autosomal_additive	associations_1e6	43

Most of the SNPs are overlapped in these five experiments, evidence that our method is effective. The false positive is low.

**Figure 3:**
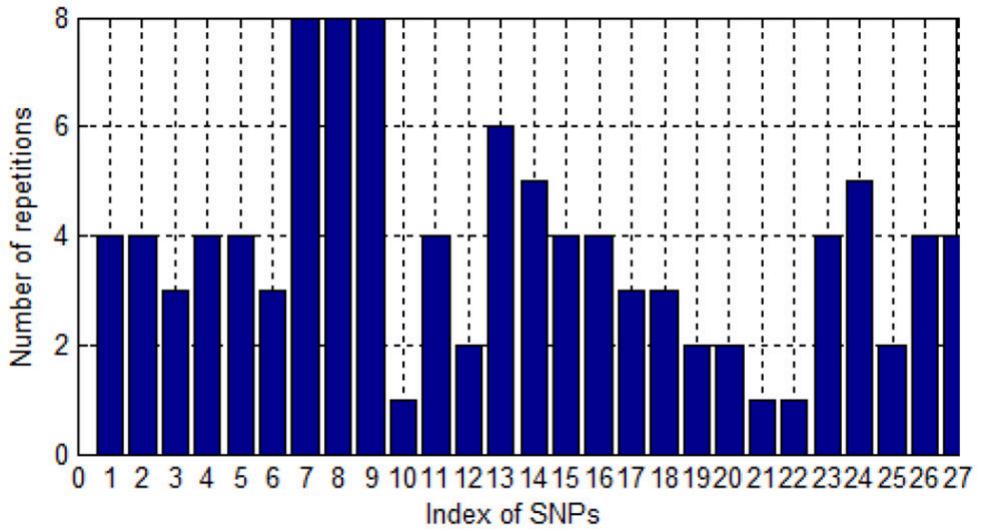
The number of repetitions of SNPs.

Since one single SNP could be labeled by different probes in the eQTL, one SNP could be screened and associated with AD several times. We collate the number of times that SNP is repeatedly screened. As shown in Figure 3, most SNPs were screened for four times. Only three SNPs were screened once. This illustrates the high accuracy, to some extent, of the SNPs screened.

**Figure 4:**
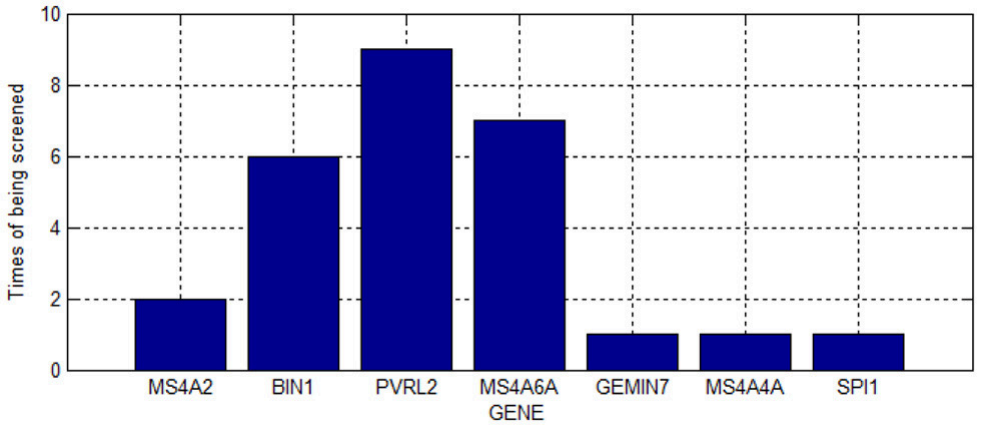
Number of gene duplication.

As shown in Table 2, we screened 27 SNPs belonging to seven genes. Most of these genes were repeatedly chosen. Figure 4 shows the number of times the genes were screened.

**Table 2:** Detailed information of selected SNP.

**Index**	**SNP**	**GENE**	***P*-value**
1	rs10402271	MS4A2	1.59e-05
2	rs4663105	BIN1	5.63e-06
3	rs11682128	BIN1	1.65e-05
4	rs6733839	BIN1	1.75e-05
5	rs10194375	BIN1	1.79e-05
6	rs56368748	BIN1	2.13e-05
7	rs6859	PVRL2	1.22e-13
8	rs377702	PVRL2	1.56e-08
9	rs157580	PVRL2	1.64e-08
10	rs439401	PVRL2	1.38e-07
11	rs8106922	PVRL2	1.31e-06
12	rs610932	MS4A6A	2.19e-06
13	rs662196	MS4A2	7.14e-06
14	rs583791	MS4A6A	7.41e-06
15	rs2075650	PVRL2	1.12e-05
16	rs676309	MS4A6A	1.14e-05
17	rs12610605	GEMIN7	1.31e-05
18	rs1562990	MS4A4A	1.39e-05
19	rs11667640	PVRL2	2.78e-05
20	rs440277	PVRL2	2.82e-05
21	rs667897	MS4A6A	3.27e-05
22	rs405509	PVRL2	6.38e-05
23	rs540170	MS4A6A	7.40e-05
24	rs581133	MS4A6A	7.92e-05
25	rs11606287	SPI1	9.49e-05
26	rs7926344	MS4A6A	9.63e-05
27	rs744373	BIN1	2.60e-05

As shown in Figure 4, PVRL2 was screened nine times. This gene is therefore very likely associated with AD.

### Gene Function

Next, we searched for known AD-related genes on different databases (as shown in Table 1) to identify which genes were novel and which genes are known.

First, we used the DisGeNET (Piñero et al., 2015) database to search for AD-related genes. In total 2244 AD-related genes were found. Three genes were identified in this database; MS4A2, MS4A6A and MS4A4A. Then, we used the ALZgene (Bertram et al., 2007) database to search for AD-related genes. Four novel genes were identified in this database; SPI1, GEMIN7, and MS4A2.

**Figure 5:**
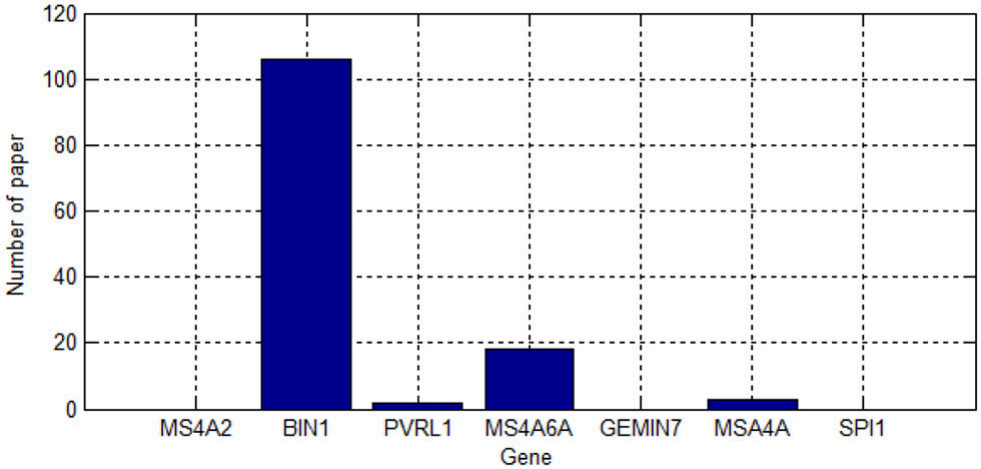
The number of papers found in the ALZgene.

We searched for the seven genes on the ALZgene database, and the number of papers found in the ALZgene are shown in Figure 5. Since MS46A and MSA4A are both included both databases, we will not discuss them in detail below. We will discuss the functions of the following five genes: BIN1, PVRL2, GEMIN7, SPI1, and MS4A2.

### BIN1

BIN1 is an encoding gene with the product name; Myc box-dependent-interacting protein 1. Several isoforms of a nucleo-cytoplasmic adaptor protein are encoded by BIN1. The isoforms expressed in the central nervous system may participate in the endocytosis of synaptic vesicles and may interact with activator proteins, synaptic proteins, endothelin and reticulin (Lee et al., 2002). Mouse studies show that this gene plays an important role in the development of myocardium. Alternatively splicing of genes leads to several transcriptional variants encoding different types of allotypes. Abnormal splicing variants expressed in tumor cell lines are also described.

Due to its ubiquitous expression in the brain, many scholars research the relationship between BIN1 and AD. Chapuis et al. (Chapuis et al., 2013) suggested that BIN1 mediates AD risk by modulating Tau pathology. Yuan et al. (Chen et al., 2018) considered that allele C at the rs744373 locus of the BINl gene was a risk factor for aMCI. The recessive model CC/CT+TT at the rs744373 locus of the BINl gene, particularly increased the risk of aMCI. Experiments were performed on 107 aMCI patients and 150 normal people.

### PVRL2

PVRL2 encodes a single pass type I glycoprotein. The protein is one component of the plasma membrane. It also acts as an entry point for the herpes simplex virus and pseudorabies virus mutants and is involved in the intercellular transmission of these viruses (Almire et al., 2010). The variation of the gene is related to the severity of multiple sclerosis.

Since PVRL2 is proven to be related to multiple sclerosis causing glial fibrils to proliferate and form calcified plaques, it is very similar to the clinical symptoms of AD. Therefore, it could be a candidate AD-related gene. Yashin et al. (2017) collected data from the Framingham Heart Study (FHS), the Cardiovascular Health Study (CHS), the Health and Retirement Study (HRS) and the Late Onset Alzheimer's Disease Family Study (LOADFS). They used logistic regression and Cox's regression and found that APOE, TOMM40, PVRL2 (NECTIN2), and APOC1 are strongly associated with AD.

### GEMIN7

The gene encodes the components of the core survival of motor neurons (SMN) complex, which plays a crucial role in the splicing pre-mRNAs in the nucleus. It has been found that the gene encodes three transcriptional variants of the same protein. It mainly expresses in the lymph nodes and the spleen.

Since, Baccon et al. (2002) discovered GEMIN7 as a new component of SMN in 2002, many researchers have studied the mechanism and protein structure of GEMIN7. SMN is a product of the disease gene of the common neurodegenerative disease spinal muscular atrophy. AD is a type of neurodegenerative disease and some patients also displayed symptoms of movement disorders. Therefore, we speculate that GEMIN7 may cause motor nerve lesions and then causes AD.

### SPI1

The ETS domain transcription factor is encoded by SPI1. It could activate gene expression during myeloid and B lymphocyte development (Zakrzewska et al., 2010). Alternative splicing of the target gene is also regulated by its protein. It broadly expresses in bone marrow.

SPI1 is expressed by microglia which are the resident immune cells of the brain. Some researches (Huang et al., 2017; Krasemann et al., 2017) suggest that PU.1, a transcription factor encoded by the gene SPI1, is a central hub in the AD gene network and is associated with AD pathology.

### MS4A2

MS4A2 (Ferreira et al., 2010) encodes the high affinity immunoglobulin epsilon receptor subunit beta, which is a member of the membrane-spanning 4A gene family. Allergic reactions involve the binding of allergens to receptor-bound IgE receptors, which are present on the surface of mast cells and basophils. Members of this new protein family share common structural characteristics and similar intron/exon splicing boundaries and exhibit unique expression patterns in hematopoietic and non-lymphoid tissues. Alternative splicing results in multiple transcript variants encoding different proteins. It has a biased expression in lung and ball bladder.

Many researches (Lambert et al., 2010; Jones et al., 2012) have found that AD is related to the overworking of the immune system. The immune system over-clears neuronal synapses, cutting off the connections between neurons, causing AD (Blasko and Grubeckloebenstein, 2003). We suggest that MS4A2 may cause abnormalities in the immune system.

## Conclusion

AD can cause progressive memory impairment, cognitive impairment, personality changes and language disorders and other neuropsychiatric symptoms, seriously affecting people's social, occupational and life functions. Identifying AD-related genes is of great clinical significance for the early diagnosis and treatment of AD.

In this paper, we used SMR to identify AD-related genes and locus. Using two GWAS datasets and five eQTL datasets, we completed ten experiments and collected effective results from a total of five experiments. In total, we identified 27 SNPs that are associated with AD. These SNPs correspond to seven genes: MS4A2, BIN1, PVRL2, MS4A6A, GEMIN7, MS4A4A, and SPI1. To verify the effectiveness of our method and the accuracy of our results, we compared the results with known databases. We found three of the seven genes in the DisGeNET database (BIN1, PVRL2, GEMIN7, and SPI1 is novel genes) and six of seven genes in the ALZGENE (SPI1, GEMIN7, and MS4A2 are novel genes). This confirms the accuracy of our results. To explain the mechanism of genetic pathogenesis, we described the function of the novel genes and speculated the mechanism of its pathogenesis.

## Author Contributions

YZ did the data preprocessing. TZh did the algorithm simulation under the direction of YH. TZa and LC helped proofreading the manuscript.

### Conflict of Interest Statement

The authors declare that the research was conducted in the absence of any commercial or financial relationships that could be construed as a potential conflict of interest.
